# Preoperative Left Ventricular Energy Loss in the Operating Theater Reflects Subjective Symptoms in Chronic Aortic Regurgitation

**DOI:** 10.3389/fsurg.2022.739743

**Published:** 2022-02-14

**Authors:** Atsushi Kainuma, Keiichi Itatani, Koichi Akiyama, Yoshifumi Naito, Maki Ishii, Masaru Shimizu, Junya Ohara, Naotoshi Nakamura, Yasufumi Nakajima, Satoshi Numata, Hitoshi Yaku, Teiji Sawa

**Affiliations:** ^1^Department of Anesthesiology, Kyoto Prefectural University of Medicine, Kyoto, Japan; ^2^Department of Cardiovascular Surgery, Kyoto Prefectural University of Medicine, Kyoto, Japan; ^3^North Medical Center, Kyoto Prefectural University of Medicine, Kyoto, Japan; ^4^Center for Mathematical Modeling and Data Science, Osaka University, Osaka, Japan; ^5^Department of Anesthesiology and Critical Care, Kansai Medical University, Osaka, Japan

**Keywords:** energy loss (EL), aortic regurgitation (AR), vector flow mapping (VFM), transesophageal echocardiography, subjective symptoms

## Abstract

**Background:**

There is currently no subjective, definitive evaluation method for therapeutic indication other than symptoms in aortic regurgitation. Energy loss, a novel parameter of cardiac workload, can be visualized and quantified using echocardiography vector flow mapping. The purpose of the present study was to evaluate whether energy loss in patients with chronic aortic regurgitation can quantify their subjective symptoms more clearly than other conventional metrics.

**Methods:**

We studied 15 patients undergoing elective aortic valve surgery for aortic regurgitation. We divided the patients into symptomatic and asymptomatic groups using their admission records. We analyzed the mean energy loss in one cardiac cycle using transesophageal echocardiography during the preoperative period. The relationships between symptoms, energy loss, and other conventional metrics were statistically analyzed.

**Results:**

There were seven and eight patients in the symptomatic and asymptomatic groups, respectively. The mean energy loss of one cardiac cycle was higher in the symptomatic group (121 mW/m [96–184]) than in the asymptomatic group (87 mW/m [80–103]) (*p* = 0.040), whereas the diastolic diameter was higher in the asymptomatic group (65 mm [59–78]) than in the symptomatic group (57 mm [51–57]) (*p* = 0.040). There was no significant difference between the symptomatic and asymptomatic groups in terms of other conventional metrics.

**Conclusions:**

An energy loss can quantify patients' subjective symptoms more clearly than other conventional metrics. The small sample size is the primary limitation of our study, further studies assessing larger cohort of patients are warranted to validate our findings.

## Introduction

There is no subjective, definitive evaluation method for therapeutic indication other than presenting symptoms in aortic regurgitation (AR). According to the 2014 American College of Cardiology/American Heart Association guidelines for the management of chronic AR, the indication for aortic valve surgery is mainly based on the presenting symptoms; the indications in asymptomatic patients are decreased and impaired systolic function or a highly dilated left ventricular chamber (1). In order not to miss the optimal timing of the surgical indication for improved postoperative patient prognosis, a novel parameter to estimate not the current cardiac function but the workload itself should be required.

Echocardiography vector flow mapping (VFM) is one of the blood flow visualization techniques which enables evaluation of cardiac energy loss (EL); these can detect the progression of cardiovascular disease (2–4). Normal pattern vortex and EL reference value of the left ventricle in adults and pediatric patients were confirmed by using VFM (5, 6). VFM analysis of valvular (7–10) and congenital heart disease (11, 12) can reportedly reveal and evaluate the effectiveness of the surgical treatment. As it does not require the use of contrast, this VFM technology can be easily used in routine clinical practice to assess ventricular vortices and predict patients' outcome (13).

Previous studies have already revealed that left ventricular diastolic EL increases proportionally to AR severity owing to turbulent vortex flow (14). However, there is still limited information about intracardiac flow evaluation in aortic valve disease (13). The aim of the present study was to determine whether left ventricular EL in chronic AR can be an alternative and superior parameter to detect symptomatic AR with surgical indication than other conventional metrics. We retrospectively analyzed patients who underwent elective surgery for severe AR with transesophageal echocardiography (TEE) under anesthesia.

## Materials and Methods

### Patients

This retrospective study was approved by the institutional review board of our institution; written informed consent was obtained from all participating patients (ERB-C-1144-1). We retrospectively analyzed patients with severe AR who underwent elective aortic valve surgery between June 2015 and December 2018. During the study period, 22 cases were detected. We excluded patients with mitral regurgitation (MR) of moderate or higher grade, coronary syndrome, intellectual disability, mild AR, emergent surgery, and adult congenital surgery (Supplementary Figure S1). Clinical data including sex, height, body weight, and body surface area, brain natriuretic peptide, and human atrial natriuretic peptide were collected on admission. We also collected patients' echocardiographic data from preoperative transthoracic echocardiography (TTE). Preoperative TTE was performed by the attending cardiologist, and all relevant echo indices were measured in accordance to the American Society of Echocardiography and the Society of cardiovascular anesthesiologist guidelines (15, 16). We divided patients into symptomatic and asymptomatic groups using their admission records. Those of stages above New York Heart Association (NYHA) class I were considered symptomatic and those at NYHA class I stage were asymptomatic. The existence of a symptom was defined according to the medical record as written by nurses and cardiovascular surgeons; these individuals were unaware of the study content.

We also enrolled one patient who was diagnosed with heart failure due to acute severe AR and underwent emergent aortic valve replacement (AVR) to obtain left ventricular vortex information. Written informed consent was obtained from this patient.

### Echocardiography

The details of the echocardiogram have been previously reported (8). After the induction of anesthesia and when the patients' vital signs were stable, TEE was performed by a TEE certified anesthesiologist (AK or KA). As EL is dependent on the measurement plane, EL estimation was performed in the fixed measurement plane for all cases, which was the left ventricular long axis view at the level of mid esophageal portion (8). We used the Aloka ProSound F75 premier ultrasound machine (Hitachi, Tokyo, Japan). Digitized 2D color Doppler cineloop images obtained in the mid-esophageal left ventricular long axis view by TEE were stored with the VFM configuration before the procedure. These images were transferred to a computer for analysis with VFM software. EL values were averaged over 3 cardiac cycles. When the aliasing phenomenon was detected, the aliasing areas were manually corrected. Measurements of systolic and diastolic EL were calculated as the mean EL during systolic and diastolic phases. The hematocrit content, the severity, the direction, and the pathology of the AR (17) and the size of the left ventricle just before the test were listed in the table of the Supplementary Table S1. TEE examination for the estimation of EL were performed before the heparinization, when patients' activated clotting time were within the normal range below 140 seconds.

### Defining Energy Loss

This technology uses both color Doppler images and speckle tracking images (2). Intracardiac energy loss can be calculated from the following equation:


$$\begin{array}{l} \text{Energy Loss}=\int \mu \left\{2{\left(\frac{\partial u}{\partial x}\right)}^{2}+2{\left(\frac{\partial v}{\partial y}\right)}^{2}+{\left(\frac{\partial u}{\partial y}+\frac{\partial v}{\partial x}\right)}^{2}\right\}dA \end{array}$$

**Figure 1:**
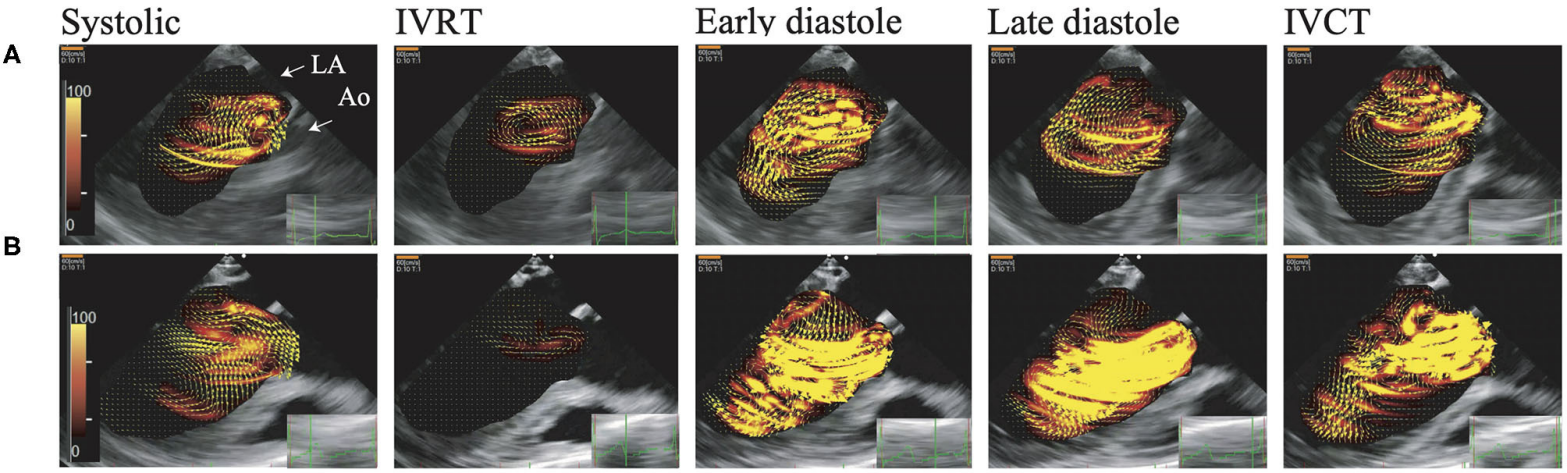
Visualization of blood flow energy loss and blood flow by vector flow mapping. These figures show EL. Brightness indicates high energy loss. The AR jet collides with mitral inflow, which causes left ventricular vortex turbulence and high dissipative EL. **(A)** Asymptomatic case (NYHA I), **(B)** symptomatic case (NYHA III). Early diastole, The early phase of the diastolic period; Systolic, Systolic phase; IVCT, The isovolumic contraction phase; IVRT, The isovolumic relaxation phase; Late diastole, The late phase of the diastolic period.

**Figure 2:**
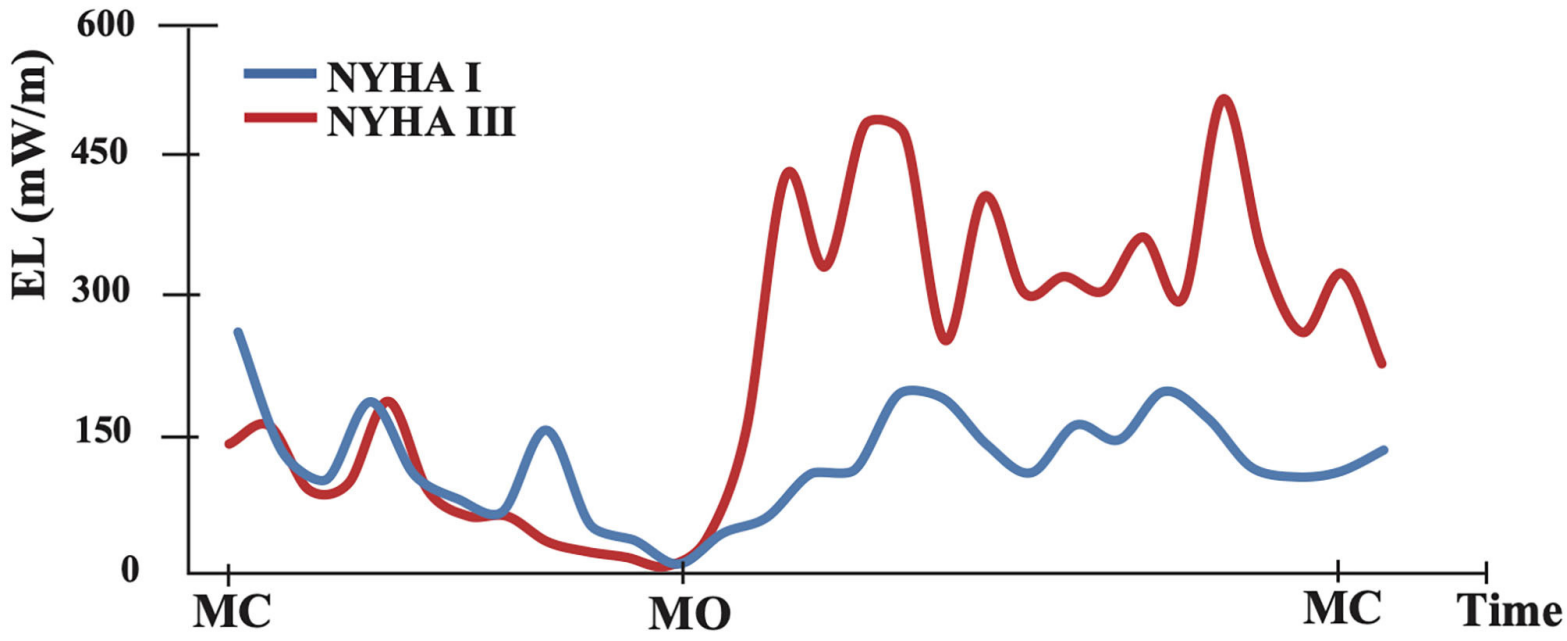
The graph shows an example of intraventricular flow energy loss distribution during one cardiac cycle. The blue curve represents the asymptomatic case (NYHA I) and red represents the symptomatic case (NYHA III). MC, mitral valve closing; MO, mitral valve opening.

Where μ is the viscosity of the blood, u and v are velocity components along the Cartesian axes (*x* and *y*), and A is the area of the unit of the grid. This is a simplified equation that is confined to 2D flow. An example of VFM analysis by TEE is shown (Supplementary Figure S2). NYHA IV and post AVR cases were not included the main analysis. Figures 1, 2 show one cardiac EL distribution in NYHA I and NYHA III cases. The main advantage of VFM is that it allows visualization of the turbulent vortex, the area where the blood flow is not laminar with disturbed direction with strong energy dissipation. EL was highest in the region of the left ventricle corresponding to the turbulence produced by the AR jet, as reported previously (14). The mean EL depends on the left ventricular preload and turbulent left ventricular vortex-like systolic anterior motion (18). Turbulent flow results in a higher EL than laminar flow, as reported previously (19). According to our previous research (6), the independent predictors of the mean EL were peak blood flow velocity at the mitral valve during early diastole (E) and heart rate (HR), with the following regression equation:


$$\begin{array}{l} \text{Mean }\text{Energy}\text{ Loss }=-46.720+0.430\times E\\ \;+0.706\times \text{Heart rate}. \end{array}$$

Our method of EL calculation is based on the same method as previously reported (6, 8, 12), and is truly reproducible and not so much beat dependent.

### Statistical Analysis

All continuous variables were skewed and summarized by the median and interquartile range. Categorical variables were summarized by frequency and percent. Continuous variables were compared between groups using Mann-Whitney *U* test. Categorical variables were compared between groups by the Fisher's exact test or chi-square test. Spearman's rank correlation was used to evaluate the correlation between EL and conventional metrics (brain natriuretic peptide, human atrial natriuretic peptide, and major echocardiographic indices). Where data were missing, we excluded them from the analysis. All analyses were performed using GraphPad Prism software (Ver.7.00, GraphPad Software, San Diego, CA USA). We defined *p* < 0.05 to indicate statistical significance.

## Results

### Participants

During the study period, 15 patients were assessed. There were seven and eight patients in the symptomatic and asymptomatic groups, respectively. There were no statistical differences between the groups' baseline characteristics (Table 1). Details of the diagnosis and symptoms on admission are shown in Supplementary Table S2.

**Table 1:** Patients' characteristics.

	**Asymptomatic group (*n =* 8)**	**Symptomatic group (*n =* 7)**	***P*-value**
Age (years)	48 [39–74]	65 [54–68]	0.694
Sex (female)	1 (12.5)	2 (28.5)	0.569
Height (cm)	174 [169–180]	165 [158–172]	0.198
Weight (kg)	70 [65–78]	62 [51–70]	0.219
Body surface area (m^2^)	1.9 [1.8–2.0]	1.7 [1.5–1.9]	0.179
**Past medical history**			
Hypertension	1 (12.5)	4 (57.1)	0.067
Chronic kidney disease	0 (0)	1 (14.3)	0.269
Dyslipidemia	0 (0)	1 (14.3)	0.269
Paroxysmal atrial fibrillation	1 (12.5)	0 (0)	0.333
Post heart failure	4 (50)	1 (14.3)	0.282
Bicuspid valve	4 (50)	1 (14.3)	0.282
NYHA class			<0.001^*^
I	8 (100)	0 (0)	
II	0 (0)	6 (85.7)	
III	0 (0)	1 (14.3)	
Other valvular disease			0.239
mild PR	0 (0)	1 (14.3)	
mild TR, mild PR	1 (12.5)	1 (14.3)	
mild TR, mild MR	1 (12.5)	4 (57.1)	
mild PR, mild MR	1 (12.5)	0 (0)	
mild TR, mild PR, mild MR	3 (37.5)	1 (14.3)	

*Data are presented as n (%) or median [IQR]*.

* *Statistically significant (p < 0.05). Abbreviations: MR, mitral regurgitation; NYHA, New York Heart Association; PR, pulmonary regurgitation; TR, tricuspid regurgitation*.

Details of the characteristics of AR in all cases are shown in Supplementary Table S1. On TEE, all patients were diagnosed with severe AR. Four patients had an AR jet directed to the center of the left ventricle, and 11 patients had a jet directed to the anterior mitral leaflet. EI Khoury I + II was the most common pathology causing AR (six cases).

An additional case was a 67-year-old man with acute severe AR causing heart failure (NYHA class 4). He was intubated and provided intensive care, but his condition did not improve, and he underwent emergency aortic valve replacement. The patient had the AR jet directed to the anterior left ventricular wall (Supplementary Figure S2). His EL decreased significantly after the procedure (Supplementary Tables S1, S2).

### Energy Loss and Vortex Pattern

**Figure 3:**
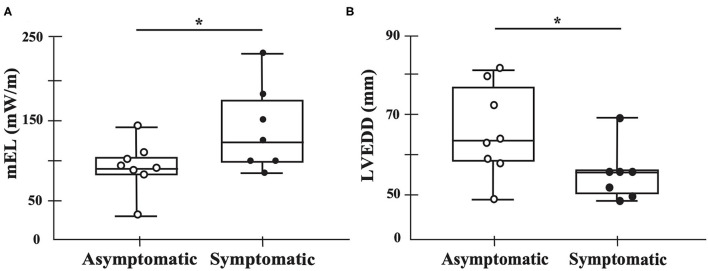
Main findings of this study. Black dots represent symptomatic patients, whereas white dots represent asymptomatic patients. The mean energy loss of one cardiac cycle is higher in the symptomatic group than in the asymptomatic group (*p* = 0.040) **(A)**. The diastolic diameter is higher in the asymptomatic group than in the symptomatic group (*p* = 0.040) **(B)**. mEL, mean energy loss in one cardiac cycle. *p < 0.05.

The mean EL of one cardiac cycle was higher in the symptomatic (121 mW/m [96–184]) than in the asymptomatic group (87 mW/m [80–103]) (*p* = 0.040), whereas the left ventricular end diastolic diameter (LVEDD) was higher in the asymptomatic group (65 mm [59–78]) than in the symptomatic group (57 mm [51–57]) (*p* = 0.040) (Figure 3; Supplementary Table S3).

We did not detect any statistically significant correlation between the symptomatic and asymptomatic groups in terms of vital signs, the hematocrit content, and the conventional and other echocardiographic data (Supplementary Figure S3; Supplementary Table S3).

### Energy Loss cut off Value

We computed a receiver operating characteristic (ROC) curve for the ability of the mean of one cardiac cycle EL to detect subjective symptoms. Our cutoff of the mean of one cardiac cycle EL value (95.5 mW/m) was associated with an increased risk of subjective symptoms in patients with severe AR (Supplementary Figure S4A). We also computed a ROC curve for LVEDD (Supplementary Figure S4B). LVEDD >58 mm in patients with chronic severe AR rarely complained about subjective symptoms in our cohort.

## Discussion

Our research suggests that left ventricular EL in chronic AR is superior to other conventional echocardiographic indices to explain pathophysiology. The main findings in the current study were as follows: (1) In severe aortic regurgitation, EL may be used to evaluate subjective symptoms more accurately than other conventional metrics; and (2) a left ventricular EL >95.5 mW/m in the mid-esophageal long axis view may be a useful diagnostic tool in symptomatic AR.

The novel point is that we investigated the relationship between EL and symptoms in patients with AR who were all in need of surgery and found that a high EL indicated patients' subjective symptoms. There were no significant differences in conventional metrics except for LVEDD, which is a main parameter for surgical indication other than subjective symptoms (1). Since all cases are diagnosed with severe AR, there was no significant difference in preload between the two groups. The reason for EL difference is thought to be vortex interaction between transmitral inflow and AR jet.

**Figure 4:**
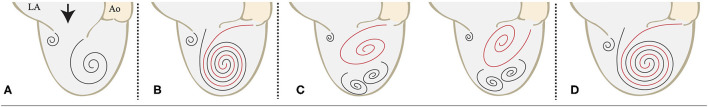
Schematics of LV vortex interaction in diastole. **(A)** The intramitral flow organizes itself in a circulatory pattern that redirects the flow to the aorta. **(B)** The AR jet collides with the transmitral flow and merges with it. **(C)** The AR jet inhibits the formation of the optimal left ventricular vortex. **(D)** The LV cavity is enlarged, and the AR jet and the transmitral flow merge. Abbreviations: AR, aortic regurgitation; LA, left atrium; Ao, aorta; LV, left ventricle.

Transmitral inflow generates vortex rings which consists of stronger anterior component and weaker posterior components (Figure 4A) (20–23). Vortex inside left ventricle, that occurs with transmitral flow known as vortex ring, not only helps smooth mitral closure, but also is believed to support efficient flow ejection toward the outlet with smooth flow turn inside left ventricle (20, 21). According to the previous imaging studies, intraventricular vortex patterns have been known to strongly associated with cardiac performance, and notably, vortex formation caused by transmitral flow plays an important role in diastolic function (24–29).

By using VFM technology, vortex changes generated by the collision of AR jet and intramitral blood flow can be visualized. Morisawa et al., reported paravalvular leakage (PVL) jet of aortic valve affects intraventricular vortex formation, and the jet direction also affects intraventricular vortex formation and vortex interaction between the transmitral flow and PVL jet (24). In our study, the left ventricular vortex was preserved in patients with NYHA 1, not well organized in patients with NYHA 2 and 3, and collapsed in patients with NYHA 4 (Supplementary Figure S2). When the AR jet merged with the transmitral flow in the same direction in diastole, the patient would be less symptomatic (Figures 4B,D). In symptomatic patients, the AR jet would interfere the consistence of left ventricular vortex ring, causing a higher EL than that noted in asymptomatic condition (Figure 4C). If the left ventricular vortex is disturbed by the AR jet, symptoms may be severe even in the absence of left ventricular enlargement, as in case 9–15 and the additional case. On the contrary, if the left ventricular vortex is still well-organized in spite of the AR jet, subjective symptoms are less likely to appear, and among these cases some cases have the left ventricle dilatation due to compensation by volume loading, as in case 1–8. As a result, asymptomatic patients with a large left ventricle are indicated for surgery. A more detailed investigation about the disturbance of left ventricular vortex by AR jet and its association with EL elevation warrant further study.

Our research finding that asymptomatic patients' diastolic diameter was higher than that in symptomatic patients reflects the fact that many asymptomatic patients underwent surgery for AR because of left ventricular enlargement. Our ROC analysis revealed that the larger the left ventricle, the less the patient complains of symptoms, if properly treated (Supplementary Figure S4B). Because they had no symptoms, the objective indications for surgery were limited; in the asymptomatic patient, progressive left ventricular dilatation and adaptation results from AR volume overload. Finally, such patients have poor cardiac function and poor surgical outcomes (30).

If we were to continue to monitor patients by using left ventricular EL, it would be easier to define the indications for surgery. Therefore, we defined a threshold value in patients with symptomatic AR. This is higher than the normal patient reference value (6). When inefficient, unphysiological flow occurs in the left ventricle, the EL rises and the heart tries to compensate. However, if the heart fails to compensate, heart failure ensues; if severe, EL may decrease owing to insufficient left ventricular energy generation (4). We, therefore, consider it necessary to evaluate patients' EL continuously.

In our study, the presence or absence of symptoms in patients was classified by the NYHA functional classification, which was determined by medical personnel not involved in this study, based on interviews and physical findings on the admission. A VO2 consumption assessment and an exercise stress echocardiography are well-known indices for quantifying symptoms (31, 32). The correlation between a VO2 consumption assessment and a stress test and the left ventricular EL needs to be assessed in the future.

Qualification of patient workload can be a good tool for treatment decision making. More large, prospective, randomized studies are needed to clarify the relationship between the progression of disease and EL. In the future, we should identify whether the AR jet causes heart failure and whether the patient has a surgical indication based on the intracardiac blood flow dynamics and EL change. The authors believe that this research demonstrates the therapeutic impact of monitoring left ventricular EL in AR.

There are several limitations to this study. First, our study is limited in that it was a retrospective, nonrandomized study assessing a relatively small cohort. Since the number of cases in the present study was so small in a single institution, future studies include accumulation of the case and follow up estimation of EL after the surgical treatment. It takes time to investigate whether EL really has a useful role in prognosis prediction. Further studies assessing a large population cohort are required to validate our findings. Second, as we primarily analyzed two-dimensional images, with software that assumed 2D flow, actual regurgitation jet is complex 3D flow, and a three-dimensional evaluation is needed to ensure accurate assessment of the severity of AR (11, 14). Three-dimensional VFM is currently unavailable. A more advanced software that can overcome this limitation needs to be developed. Third, these data were analyzed by TEE and were from patients who were under general anesthesia. TEE provide superior visualization of the left ventricle without the intervening lung and bone compared with TTE. There is modest agreement in AR assessment between TTE and intraoperative TEE (33). However, there is limited information on the difference between TEE and TTE in terms of left ventricular EL analysis. Since TTE is also important in clinical practice, it is necessary to analyze TTE data in the future. Fourth, all patients in this research were already considered suitable to undergo surgery; we should also analyze patients who are not. Fifth, anesthetic agents may affect viscosity, which would alter the EL cutoff values. Examination of the role of this parameter as a predictive one of the prognoses of the left ventricular function warrants further study including the follow up EL with trans thoracic echocardiography of these patients.

## Conclusions

In summary, EL calculated by VFM can quantify patients' subjective symptoms more accurately than other conventional metrics in chronic AR. VFM can visualize the interference of the AR jet with intra-mitral flow and alteration of the intraventricular vortex direction, which increase the cardiac workload. Large prospective studies are necessary to further assess the utility of this technique for patients with AR.

## Data Availability Statement

The data that support the findings of this study are available on request from the corresponding author, due to privacy/ethical restrictions.

## Ethics Statement

The studies involving human participants were reviewed and approved by Kyoto Prefectural University of Medicine. The patients/participants provided their written informed consent to participate in this study.

## Author Contributions

AK made substantial contributions to conception and design, acquired and analyzed data, performed the statistical analysis, and drafted the manuscript. KI made substantial contributions to the conception and design, acquiring and analyzing data, performing the statistical analysis, and drafting the manuscript. KA made substantial contributions to acquiring and analyzing data. YNai made substantial contributions to acquiring the data. MI made substantial contributions to the conception and design and revised the manuscript for intellectual content. MS and JO made substantial contributions to acquiring and analyzing the data. NN made substantial contributions to performing the statistical analysis and revised the manuscript for intellectual content. YNak made substantial contributions to analyzing data and revised the manuscript for intellectual content. SN, HY, and TS made substantial contributions to the conception and design, drafting the manuscript, and revising the manuscript for intellectual content. All authors read and approved the final manuscript.

## Funding

This work was supported by the Japan Society for the Promotion of Science and a Grant-in-Aid for Scientific Research KAKENHI to MI (No. 16K10970) from The Ministry of Education, Culture, Sports, Science and Technology, Japan.

## Conflict of Interest

KI is an equity shareholder and founder of the vendor for the blood flow analysis tool: Cardio Flow Design Inc. Tokyo, Japan and an endowed chair of Kyoto Prefectural University of Medicine, financially supported by Medtronic Japan (Tokyo, Japan). The remaining authors declare that the research was conducted in the absence of any commercial or financial relationships that could be construed as a potential conflict of interest.

## Publisher's Note

All claims expressed in this article are solely those of the authors and do not necessarily represent those of their affiliated organizations, or those of the publisher, the editors and the reviewers. Any product that may be evaluated in this article, or claim that may be made by its manufacturer, is not guaranteed or endorsed by the publisher.
