# Supplementary material for: Preoperative Left Ventricular Energy Loss in the Operating Theater Reflects Subjective Symptoms in Chronic Aortic Regurgitation
Source: Front Surg. 2022 Feb 14;9:739743. doi: 10.3389/fsurg.2022.739743 (PMC8889468; doi:10.3389/fsurg.2022.739743)
Supplement: Supplementary file 1 [file Data_Sheet_1.doc]

**SUPPLEMENTARY MATERIAL**

| Table of Contents |  | **Page** |
| --- | --- | --- |
| **Supplementary Figure S1:** **Study flow chart.**  **Supplementary Figure S2:** **Visualization of blood flow by vector flow mapping.**  **Supplementary Figure S3:** **Correlations between mean energy loss in one cardiac cycle and other parameters.**  **Supplementary Figure S4:** **Receiver operating characteristic curve for revealing association between mEL, LVEDD, and patient subjective symptoms.**  **Supplementary Table S1:** **Details of the characteristics of aortic regurgitation in all cases.**  **Supplementary Table S2:** **Details of the diagnosis and symptoms on admission.**  **Supplementary Table S3:** **Study results.** |  | 2  3  4  5  6  8  10 |

**Supplementary material, Figure 1:** Study flow chart.

* We enrolled patients who was diagnosed heart failure caused by acute severe AR and underwent emergent surgery to obtain left ventricular vortex information.

**
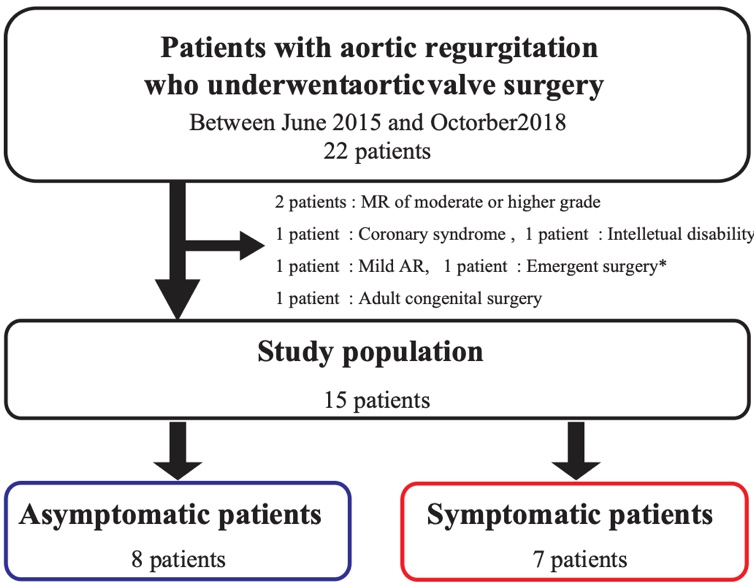
**

**Supplementary material, Fig 2:** Visualization of blood flow by vector flow mapping.

Intraventricular vector flow mapping in the patient at the stages NYHA I (Asymptomatic), II-IV (Symptomatic), and post AVR. E) Early diastole phase. L) Late diastole phase. Abbreviations: Ao, Aorta, LA, left atrium.


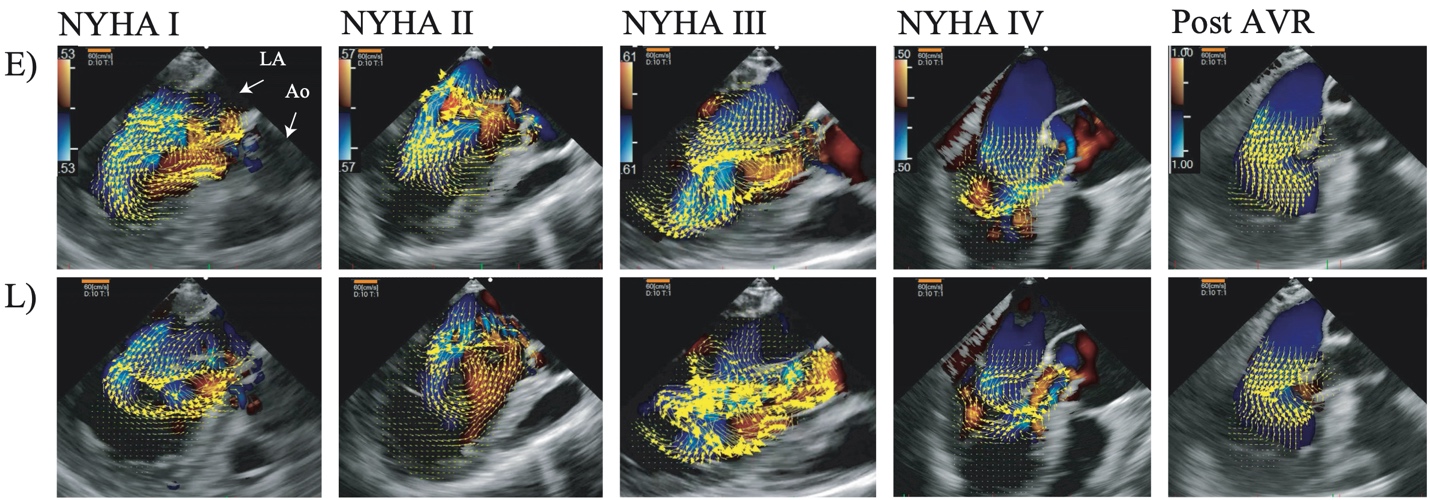


**Supplementary material, Figure 3:** Correlations between mean energy loss in one cardiac cycle and other parameters.

There are no statistical correlations between the mean energy loss in one cardiac cycle and other parameters. a) mEL and brain natriuretic peptide (BNP). b) mEL and human atrial natriuretic peptide (hANP). c) mEL and left ventricular ejection fraction (LVEF). d) mEL and LVEDD. e) mEL and left ventricular end-systolic diameter (LVESD). f) mEL and regurgitation volume (RV). g) mEL and pressure half time (PHT). h) mEL and effective regurgitant orifice (ERO). i) mEL and Vena Contracta (VC).

**
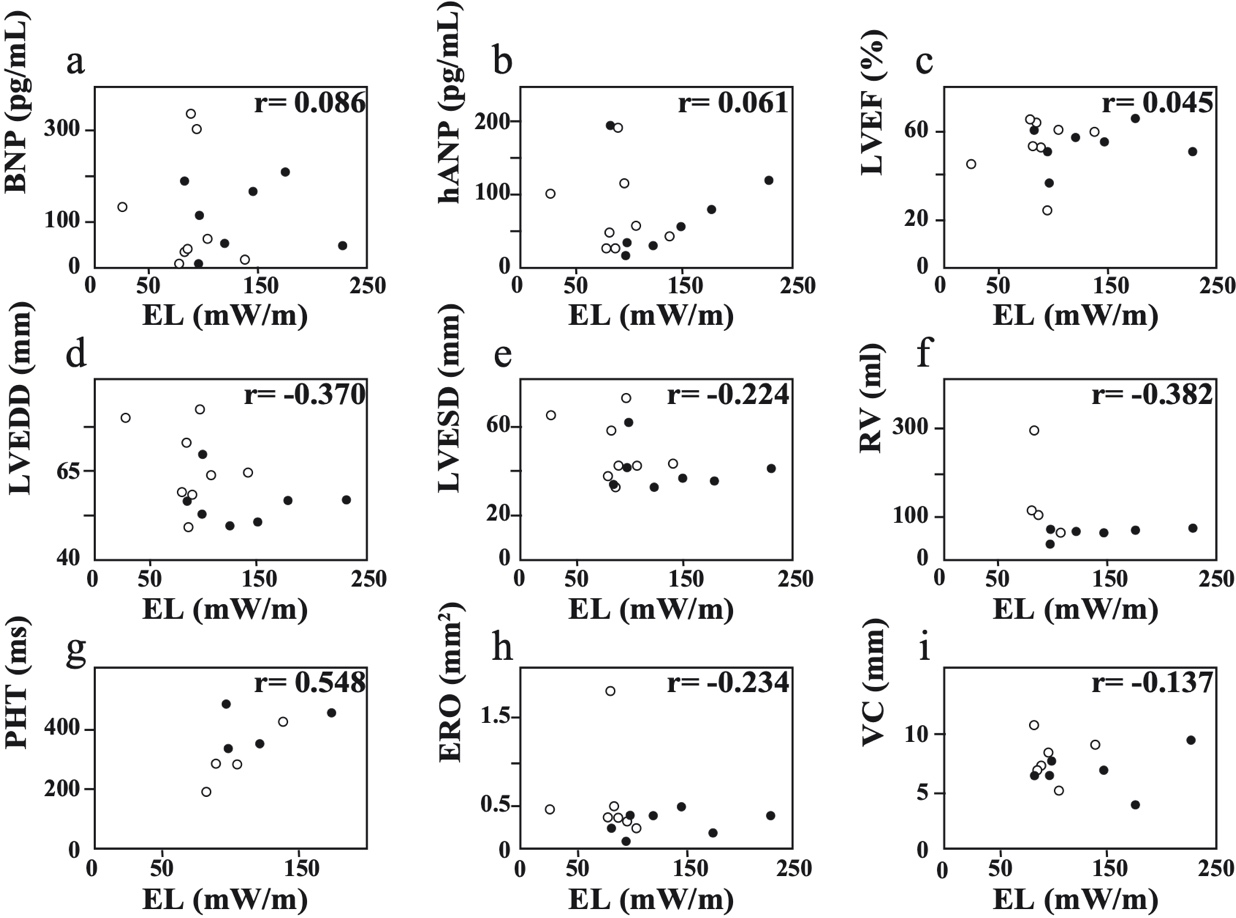
**

**Supplementary material, Figure 4 A and B:** Receiver operating characteristic curve for revealing association between mEL, LVEDD, and patient subjective symptoms.

Our cutoff value (mEL = 95.5 mW/m, LVEDD = 58mm) is that value which corresponds to a point on the receiver operating characteristic curve nearest to the upper corner of the graph. Abbreviations: AUC, area under curve.

**
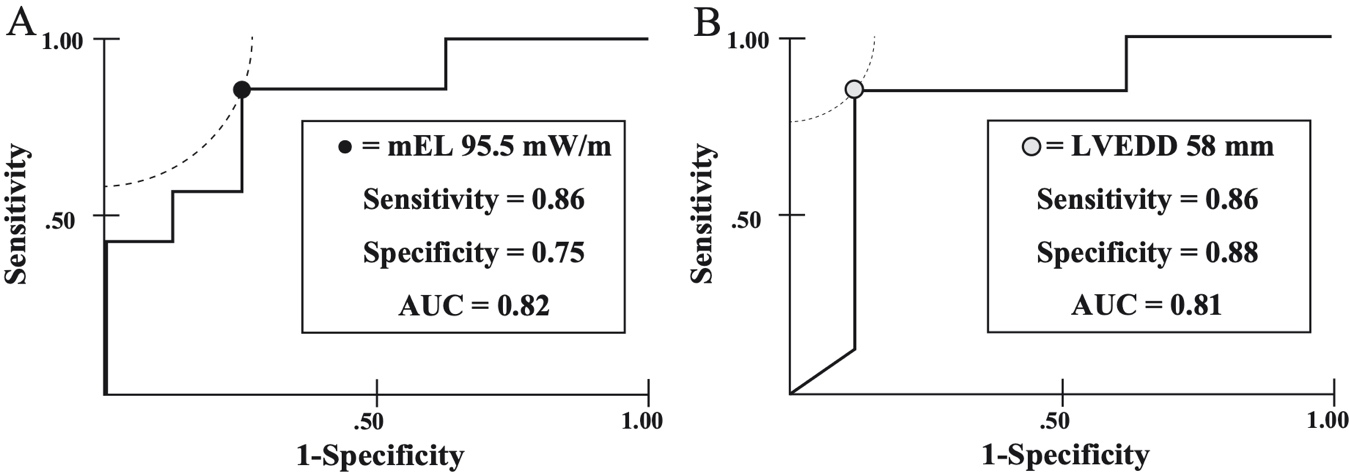
**

**Supplementary material, Table S1. Details of the characteristics of aortic regurgitation in all cases.**

| ID | NYHA | The pathology causing AR | The direction of the AR jet | Hematocrit (%) | LVEDD (mm) | LVESD (mm) | LVEF (%) | EL  (mW/m) |
| --- | --- | --- | --- | --- | --- | --- | --- | --- |
| 1 | 1 | El Khoury type Ⅱ (RL fusion BAV) | Anterior mitral valve | 41 | 60 | 38 | 65 | 79 |
| 2 | 1 | El Khoury type Ⅰ (RL fusion BAV) + Ⅱ | Central jet | 52.9 | 82 | 74 | 25 | 95 |
| 3 | 1 | El Khoury type Ⅰ + Ⅱ | Anterior mitral valve | 32.8 | 64 | 43 | 60 | 105 |
| 4 | 1 | EI Khoury Ⅰ + Ⅱ | Anterior mitral valve | 38.7 | 80 | 66 | 45 | 27 |
| 5 | 1 | EI Khoury Ⅰ | Anterior mitral valve | 38.8 | 59 | 43 | 52 | 89 |
| 6 | 1 | EI Khoury Ⅱ | Anterior mitral valve | 39.2 | 50 | 33 | 63 | 85 |
| 7 | 1 | El Khoury type Ⅰ (RL fusion BAV) + Ⅱ | Central jet | 44.9 | 73 | 59 | 54 | 82 |
| 8 | 1 | El Khoury type Ⅰ (NR fusion BAV) +Ⅱ | Anterior mitral valve | 51 | 65 | 44 | 59 | 138 |
| 9 | 2 | EI Khoury Ⅲ | Central jet | 33 | 57 | 36 | 66 | 184 |
| 10 | 2 | EI Khoury Ⅱ | Anterior mitral valve | 38.4 | 50 | 33 | 57 | 121 |
| 11 | 2 | El Khoury type Ⅰ | Anterior mitral valve | 45.1 | 57 | 34 | 61 | 83 |
| 12 | 2 | El Khoury type Ⅰ (RL fusion BAV) + Ⅱ | Anterior mitral valve | 43.2 | 53 | 42 | 51 | 96 |
| 13 | 3 | El Khoury type Ⅰ | Anterior mitral valve | 31.9 | 57 | 42 | 51 | 228 |
| 14 | 2 | El Khoury type Ⅰ | Anterior mitral valve | 45.3 | 70 | 63 | 37 | 98 |
| 15 | 2 | EI Khoury Ⅲ | Central | 34.3 | 51 | 37 | 55 | 147 |
| E | 4 | EI Khoury Ⅱ, NCC prolapse | Anterior left ventricular wall | 38.4 | 54 | 36.8 | 39 | 109 |
| E post | n/a | n/a | n/a | n/a | n/a | n/a | n/a | 18.7 |

Abbreviations: AR, aortic regurgitation; AV, aortic valve; BAV, bicuspid valve; LVEDD, left ventricular end-diastolic diameter; LVEF, left ventricular ejection fraction; LVESD, left ventricular end-systolic diameter; NCC, non-coronary cusp; RL, right-left; NR, non-right.

**Supplementary material, Table S2. Details of the diagnosis and symptoms on admission.**

| ID | NYHA | Gender | Age | Preoperative Diagnosis | Types of valve | Symptoms on admission, comments |
| --- | --- | --- | --- | --- | --- | --- |
| 1 | 1 | Male | 31 | Aortic regurgitation | Bicuspid | Asymptoms, pointed out AR in workplace examination |
| 2 | 1 | Male | 43 | Aortic regurgitation | Bicuspid | Asymptoms, after treatment for heart failure |
| 3 | 1 | Female | 78 | Aortic regurgitation, Ascending aortic aneurysm | Tricuspid | Asymptoms, enlargement of the ascending aorta |
| 4 | 1 | Male | 53 | Aortic regurgitation, Annuloaortic ectasia, Marfan syndrome | Tricuspid | Asymptoms, after treatment for heart failure |
| 5 | 1 | Male | 72 | Aortic regurgitation, Annuloaortic ectasia | Tricuspid | Asymptoms, after treatment for heart failure |
| 6 | 1 | Male | 79 | Aortic regurgitation | Tricuspid | Asymptoms, after treatment for heart failure |
| 7 | 1 | Male | 33 | Aortic regurgitation | Bicuspid | Asymptoms |
| 8 | 1 | Male | 41 | Aortic regurgitation, Ascending aortic aneurysm | Bicuspid | Asymptoms, enlargement of the ascending aorta |
| 9 | 2 | Male | 84 | Aortic regurgitation | Tricuspid | Exertional dyspnea |
| 10 | 2 | Male | 65 | Aortic regurgitation | Tricuspid | Exertional palpitation |
| 11 | 2 | Male | 70 | Aortic regurgitation, Annuloaortic ectasia, Ascending aortic aneurysm | Tricuspid | Exertional dyspnea |
| 12 | 2 | Male | 28 | Aortic regurgitation | Bicuspid | Exertional dyspnea |
| 13 | 3 | Female | 49 | Aortic regurgitation | Tricuspid | Exertional chest pain |
| 14 | 2 | Male | 59 | Aortic regurgitation, Annuloaortic ectasia | Tricuspid | Exertional dyspnea |
| 15 | 2 | Female | 66 | Aortic regurgitation | Tricuspid | Exertional dyspnea |
| E | 4 | Male | 67 | Aortic regurgitation | Tricuspid | Acute heart failure, Emergent surgery |
| E post |  | Male | 67 | Post aortic valve replacement | Prosthetic valve | Acute heart failure, Post aortic valve replacement |

**Supplementary material, Table S3. Study results.**

|  | Asymptomatic group (n=8) | Symptomatic group (n=7) | *P* value |
| --- | --- | --- | --- |
| HR (/min) | 61 [49 to 63] | 61 [54 to 63] | 0.843 |
| sBP (mmHg) | 98 [86 to 103] | 121 [104 to 128] | 0.051 |
| dBP (mmHg) | 37 [32 to 45] | 45 [37 to 50] | 0.480 |
| BNP (pg/mL) | 51 [30 to 178] | 116 [52 to 181] | 0.778 |
| h-ANP (pg/mL) | 52 [41 to 106] | 59 [33 to 101] | 0.955 |
| Hematocrit (%) | 40.1 [39 to 49] | 38 [33 to 45] | 0.385 |
| Echocardiographic data |  |  |  |
| mEL (mW/m) | 87 [80 to 103] | 121 [96 to 184] | 0.040* |
| LVEF (%) | 57 [47 to 62] | 55 [51 to 61] | 0.930 |
| LVEDD (mm) | 65 [59 to 78] | 57 [51 to 57] | 0.040* |
| LVESD (mm) | 44 [40 to 64] | 37 [34 to 42] | 0.100 |
| RV (ml/beat) | 104 [90 to 153] | 65 [63 to 68] | 0.171 |
| PHT (ms) | 288 [263 to 324] | 409 [351 to 469] | 0.114 |
| ERO (mm^2^) | 0.39 [0.35 to 0.49] | 0.34 [0.24 to 0.42] | 0.301 |
| VC (mm) | 7.4 [6.7 to 8.5] | 6.4 [6.1 to 7.1] | 0.331 |

Data are presented as n (%) or median (IQR). Abbreviations: BNP, brain natriuretic peptide; dBP, diastolic blood pressure; h-ANP, human atrial natriuretic peptide; HR, heart rate; LVEDD, left ventricular end-diastolic diameter; LVEF, left ventricular ejection fraction; LVESD, left ventricular end-systolic diameter; mEL, mean energy loss in one cardiac cycle; sBP, systolic blood pressure. *: p < 0.05
